# Systematic Literature Review of IoT Botnet DDOS Attacks and Evaluation of Detection Techniques

**DOI:** 10.3390/s24113571

**Published:** 2024-06-01

**Authors:** Metehan Gelgi, Yueting Guan, Sanjay Arunachala, Maddi Samba Siva Rao, Nicola Dragoni

**Affiliations:** DTU Compute, Technical University of Denmark (DTU), 2800 Kongens Lyngby, Denmark; s231846@dtu.dk (Y.G.); s232884@dtu.dk (S.A.); s230010@dtu.dk (M.S.S.R.)

**Keywords:** IoT, DDoS, botnet, botnet attacks, detection

## Abstract

Internet of Things (IoT) technology has become an inevitable part of our daily lives. With the increase in usage of IoT Devices, manufacturers continuously develop IoT technology. However, the security of IoT devices is left behind in those developments due to cost, size, and computational power limitations. Since these IoT devices are connected to the Internet and have low security levels, one of the main risks of these devices is being compromised by malicious malware and becoming part of IoT botnets. IoT botnets are used for launching different types of large-scale attacks including Distributed Denial-of-Service (DDoS) attacks. These attacks are continuously evolving, and researchers have conducted numerous analyses and studies in this area to narrow security vulnerabilities. This paper systematically reviews the prominent literature on IoT botnet DDoS attacks and detection techniques. Architecture IoT botnet DDoS attacks, evaluations of those attacks, and systematically categorized detection techniques are discussed in detail. The paper presents current threats and detection techniques, and some open research questions are recommended for future studies in this field.

## 1. Introduction

‘Internet of Things’ (IoT) is a network combining physical devices with communication and sharing information [1]. The physical devices include all electronic devices such as phones, and computers but also smart home appliances and industrial sensors. IoT changes our daily lives; however, one of the huge vulnerabilities of this interconnection is Distributed Denial of Service (DDoS) attacks.

A DDoS attack is a malicious attempt to destroy the normal traffic of a targeted server, service, or network by overwhelming the target or its surrounding infrastructure with a flood of Internet traffic. DDoS attacks can severely damage networks and disrupt services, leading to significant economic and operational impacts. According to a report by Netscout [2], the frequency and intensity of these attacks have increased in 2023, rising from an average of 144 daily attacks at the start of the year to 611 by the end of June, an increase of approximately 353%. Cloudflare reported a 67% increase in ransom DDoS attacks in 2022, highlighting a trend towards financially motivated cybercrimes. These data points underscore the substantial impact and evolving nature of DDoS threats in the digital landscape [3].

IoT devices are frequently exploited as tools in cyberattacks, without the owners’ awareness. These devices can be hijacked and added to a network of infected devices, known as a ‘botnet’. These botnets are networks of private computers infected with malicious software and controlled as a group, commonly used to carry out DDoS attacks. The role of IoT in these botnets is increasingly alarming due to the often inadequate security measures in these devices. The 2022–2023 IoT Botnet Report by CUJO AI [4] highlights the increasing exploitation of vulnerabilities in IoT devices for botnet activities, demonstrating the critical role of IoT botnets in DDoS attacks. This growing trend underscores the need for enhanced security measures in IoT devices to mitigate the risk of such cyberattacks.

This literature review comprehensively analyzes the current state of IoT botnet-induced DDoS attacks. It seeks to understand the architecture of these botnets, evaluate the methodologies used in such attacks, and review the detection techniques proposed in recent literature. By focusing on these aspects, the review aims to highlight the vulnerabilities inherent in IoT devices, assess the effectiveness of current detection strategies, and identify areas that need further research and development to strengthen IoT security against DDoS attacks. Mitigation strategies for IoT botnet DDoS attacks are excluded from this literature review due to the extensive research already conducted in this area. This exclusion allows for a more focused analysis of detection techniques and the architecture of IoT botnets, areas where further research is critically needed.

### 1.1. Contribution of This Paper

In this literature paper, we aimed to provide an up-to-date literature review of DDoS attacks and detection techniques focused on IoT botnets. Compared to previous literature reviews, this paper covers specific research focuses, which are summarized in Table 1. In this literature review, the main contributions can be summarized as follows:

- Focus on various DDoS Attacks of IoT botnets and detailed architecture of botnet attacks;
- Analysis of IoT botnet attacks on an evaluative basis;
- Discussion of different detection techniques, including ML/DL solutions, to offer a comprehensive overview of the available solutions;
- Proposal of a taxonomy of IoT botnet DDoS attacks and detection techniques;
- Listing of current threats and most recent detection techniques;
- Discussion of open questions and future research in this increasingly crucial domain.

### 1.2. Outline of This Paper

The rest of the paper is organized as follows. In Section 2, we discuss and summarize other surveys/literature reviews, and we highlight the novelty of this paper. In Section 3, we describe the research methodology, the research questions, and the inclusion and exclusion criteria used to identify the papers to be reviewed. In Section 4, we cover various IoT botnets and related architectures that are used to create such botnets. In Section 5, we discuss the IoT botnet evolution through the years. In Section 6, we deep dive into DDoS detection techniques, including traditional and latest detection techniques. In Section 7, we briefly discuss the emerging IoT botnet DDoS attacks and newly developed detection systems that are effective for the latest modified botnets. In Section 8 and Section 9, we briefly discuss the main findings and open questions/future work, respectively. Section 10 concludes the paper with specifying contributions to the literature.

## 2. Related Work

IoT and DDoS attacks are popular in the literature, which has resulted in extensive research in this area with varied scope and focus. Table 1 gives an overview of related works that analyze different aspects of this research area.

Thanh et al. [13] have conducted one of the most comprehensive literature reviews in this field in recent years. The survey conducts a detailed literature research, and with 234 references, it has performed quite a deep analysis of the research field. It focuses on botnets from different perspectives, which include the architecture and evaluation of botnet attacks, and also gives detection techniques for corresponding attacks. Stephens et al. [12] have conducted comparative research on IoT botnets. It is a well-structured literature review that includes a systematic review of recent IoT botnet detection and mitigation literature (2015–2020). A comparative study is well-designed with qualitative and quantitative comparisons. This paper also includes emerging threats and detection techniques to leave open questions about these research areas. Vishwakarma et al. [9] discuss security issues in IoT networks, focusing on DDoS attacks in this domain. The paper also explains attacks and their impacts with data to demonstrate the evaluation of attacks.

Many surveys in botnets focus on an overview of attack architectures and explain different types of attacks as can be seen in Table 1. However, these papers cannot give enough emphasis on botnet DDoS attacks. In contrast, Vishwakarma et al. [9] focus on DDoS attacks in botnets and offer a comprehensive overview for researchers.

Feily et al. [5] and Silva et al. [6] give an overview of IoT botnets and their architectures of attacks with impacts. These papers and some other earlier papers, such as [7,8], have a limited number of detection techniques available and do not group detection techniques as host-based or network-based solutions. They mainly focus on individual detection techniques without providing taxonomies.

The advancements in machine learning solutions have also impacted IoT botnet detection systems. More studies are conducted in this field with the development of ML-based solutions. ML- and DL-based botnet detection techniques are first mentioned in [9] within the literature review papers in this area. Subsequently, ML/DL solutions became the most prominent detection technique parts. As a result, most recent papers in the literature focus on deep learning. In recent years, some literature reviews, such as [17,21] have only focused on deep learning-based detection systems. In parallel, different methods have continued to be developed to detect botnet DDoS attacks. Blockchain-based [18,22,23] and SDN [24,25] based solutions have also started to become popular, which have resulted in more research focus in this field. Some papers are revolved around blockchain-based detection techniques. Shah et al. [18] claim that their study is the first literature review that focuses on DDoS attacks in IoT environments that use the blockchain.

On top of that, some literature review papers [13,14,16,17] provide a well-defined search strategy, which contributes to a more systematic way of analyzing literature. These sections give a road map for other researchers to analyze literature effectively.

## 3. Methodology

### 3.1. Systematic Literature Review Strategy

This section explains the comprehensive research strategy employed in conducting the systematic literature review for this study.

#### 3.1.1. Research Questions

The research questions asked by this paper are as follows:

1. What are the IoT botnets DDoS attacks, their evaluations, and their impacts?
2. What are the current IoT botnet threads?
3. What are the state-of-the-art IoT botnets DDoS detection mechanisms?
4. What are the methodologies, strengths, and weaknesses of existing approaches?
5. What are the current IoT botnet threads and detection mechanisms developed in the recent research?

#### 3.1.2. Search Strategy

This section describes the search strategy of this paper and explains the methodologies used to select the literature for this paper. In this literature review, strategies are followed based on guidelines from Petersen [26] and Wohlin [27]. These methodologies provide extended guidelines for systematic literature review. These guidelines explain the research strategy for analyzing literature and the snowballing strategy for sampling papers with inclusion and exclusion techniques. In Table 2, an overview of paper selection steps is given. In the study selection process section, each step is explained in detail.

#### 3.1.3. Study Selection Process

This study aimed to find answers to the research questions (Section 3.1.1) specific to IoT botnet DDoS attacks and detection techniques. In the scope of this paper, mitigation strategies are excluded since research in that area is also crucial and should be analyzed in detail. For this purpose, this study starts with source selection for literature research and DTUFindIt is chosen as a paper research source since it is accessible by DTU account and provides access to the full papers. Research of the literature is started with a query including some keywords from this study. As a first step, 1125 papers are found with a given query in Table 2. Then, this query is extended to cover research questions, and as a result of this query, 328 papers are found.

After obtaining these papers, several exclusion and inclusion steps are applied to identify the required papers for this research. An initial exclusion is executed according to some basic criteria: only fully accessible from DTU inside freely; English; peer reviewed; and IoT botnet DDoS-related papers. After applying these filters, 300 papers are left.

After the initial exclusion, the authors analyze the papers’ titles/abstracts to exclude irrelevant papers and include papers only related to the research questions. Specifically, papers are selected if they address and answer at least three out of the five research questions introduced in Section 3.1.1. After this step, 144 papers are left. Of these, 28 papers are literature review papers that are written on this topic. It shows that this topic is popular in the literature. As explained in Section 1.1 one of the contributions of this paper is to analyze different literature review papers and report different IoT botnet DDoS attacks and detection approaches on an evaluative basis.

An intermediate backward snowballing step is applied. In this step, more queries are searched in DTUFindIt to include missing papers due to the initial query. Some of these queries are “(Botnet AND Detection), (“IOT Botnet” AND Deep Learning)…”. In addition to these queries, other external papers are added, which are found by individual searches.

Final full-text reviews are performed for the 183 papers found. Each author is assigned some papers and reviews them to create a shortlist of papers with details included. For the full-text reviews, our main criteria are the research questions. We try to select papers that answer the research questions and are focused on IoT botnet DDoS attacks and/or detection techniques. This strategy allows each author to understand the details of the papers with a target focus. At this point, 102 papers remain to be included in this literature review.

As a final step, some supporting papers/resources are added during the process of writing the literature review to better answer the research questions.

## 4. Iot Botnets and Architectures

Understanding the intricacies of IoT botnet architectures is crucial for developing effective strategies to protect against evolving cyberthreats in the connected world.

**Figure 1:**
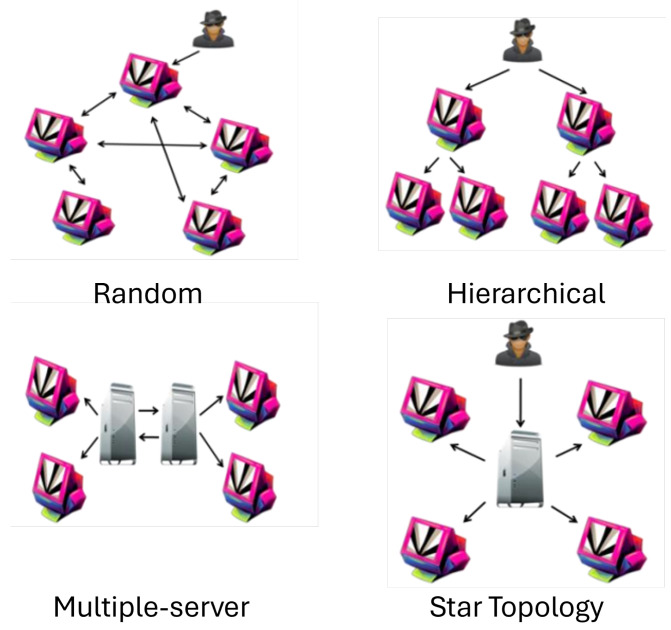
Botnet architecture [30].

The architecture of a botnet is classified into four types: star topology, multiple-server, hierarchical, and random topology [28,29]. The most popular and quickly infecting type of botnet is the centralized botnet, often known as star topology as seen in Figure 1. When a bot master posts a command to the control-and-command server, the server distributes the command to all the bots, initiating an attack. The attack will begin with the attack pattern that the bot master has created once the bots receive the command. The control-and-command server, which forms the basis of this architecture, can be located and used by an Internet service provider or researcher to effectively take down a botnet. The bots cannot receive commands from the bot master if the connection between the control-and-command servers is blocked, which will prevent the attack from succeeding [30]. In many server topologies, the number of control-and-command servers is different from the star topology. The configurations of the control-and-command servers are altered by the many server topology because of how easily things can go wrong. Each of the connected control-and-command servers is configured to post commands. When one of the servers is detected and breaks down, another server will take its place, ensuring that the botnet continues to function as intended. The attack will continue as long as one of the command-and-control servers is active according to the bot master [30]. The multiple-server architecture has certain drawbacks. Multiple-server botnet construction is considered more difficult by the bot master because of its complexity compared to a star topology. A control-and-command server is not required in the hierarchical botnet as depicted in Figure 1 because it contains multiple high-level bots. To make the C&C server and bot master more hidden, high-level bots are employed as a C&C server. As a result of the C&C server’s protection, if the bot master builds the botnet utilizing a hierarchical architecture, it is difficult to destroy [30]. If the high-level bot is located, the botnet only loses a portion of its bot population. Figure 1 depicts a random botnet’s architecture. The random botnet lacks the command-and-control server as seen in Figure 1. One bot will communicate commands to other bots connected to it whenever it receives them from the bot master. Despite being extremely difficult to construct, a random botnet has good security because each bot is interpreted as a C&C server [31]. A key problem with the centralized botnet is identifying and taking down the C&C servers. The C&C server in the P2P botnet is extremely difficult to find because each bot serves as a C&C server, so if one of the bots in a random topology botnet’s architecture is discovered, its impacts are limited and cannot bring down the entire network [32].

Common components of IoT botnet architecture are categorized into three key elements:

1. **Infected devices:** The infected devices are the core of any Internet of Things botnet. These gadgets can include thermostats, smart refrigerators, and security cameras in addition to routers.
2. **Command-and-control (C2) servers:** The infected devices receive instructions from the C2 servers, which function as orchestrators, coordinating their actions. To avoid being discovered, these servers are frequently hosted on the dark web.
3. **Propagation mechanisms:** IoT botnets propagate using a variety of techniques, such as using malware droppers, brute force attacks on default credentials, and weaknesses in IoT device firmware [30].

Mirai botnet is one of the most encountered and powerful botnets. Much research has been conducted on Mirai to understand it better and to come up with strong detection techniques. The below section explains the components specific to Mirai botnet and its attacking strategies.

### Mirai Botnet Components

**Figure 2:**
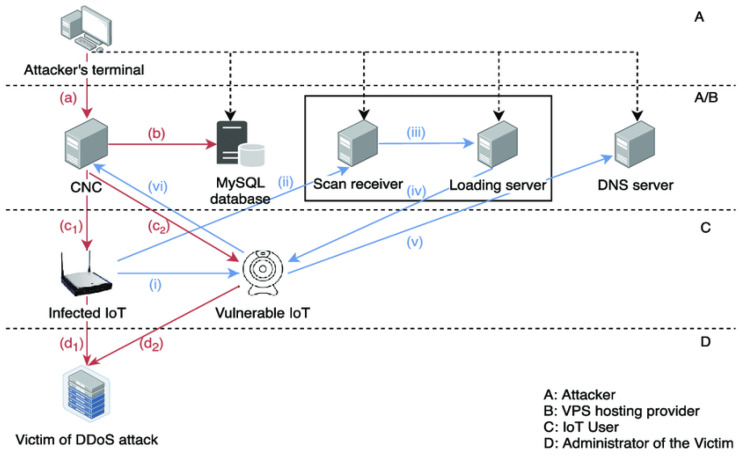
Mirai botnet architecture [33].

According to the Mirai source code [33], a typical Mirai botnet consists of a command-and-control (CNC) server, a MySQL database server, a Scan Receiver, a loading server (also known as a Loader), and a DNS server. A DDoS attack can be initiated by an attacker by delivering a specific command via Telnet from a remote terminal to the CNC server (step a), as Figure 2 illustrates. The instructions are simultaneously recorded on the MySQL database server (step b). In step c1, the attack target is routed to the compromised IoT devices (or bots). The intended victim server receives a flood of network packets from live bots, which then comply with the CNC command (step d1).

Furthermore, an infected IoT device can search the network from a variety of IP addresses for other susceptible IoT devices (step I). The bot notifies the Scan Receiver (step II) of any discovery of a susceptible device, along with its IP address, user credential, type of service, etc. The Loader proactively gathers information about the vulnerable device as soon as a new report is received. The reason the Scan Receiver and the Loader were thought to be on the same machine in this case is illustrated in Figure 2. By default, the Scan Receiver adds the information about the vulnerable device to the operating system’s standard output stream, or stdout, which is constantly being watched over by the Loader (step III) [33].

The malware is then uploaded by the Loader after logging into the susceptible device (step IV). The newly infected IoT device then is configured as a new bot, which needs to register with the CNC server (step VI). Before this stage, the susceptible device needs to obtain the CNC server’s IP address from a DNS server that is hardcoded (step v). The identical circumstance arises when an infected device wants to connect with the Scan Receiver. Due to this design, an attacker can shift the IP address of every other server to a new one as long as the DNS server is operational [33].

## 5. Evolution of IoT Botnets

The growth of the Internet of Things (IoT) is always combined with widespread vulnerabilities and has always attracted malicious actors. The emergence of Internet Relay Chat (IRC) in the late 1990s gave rise to the notion of botnets [34]. Cybercriminals used IRC channels as a means of generating botnet networks of compromised computers. Usually, these bots were employed for illegal activities like spamming and denial-of-service (DDoS) attacks. Early botnets were rather simple, operating on straightforward commands and scripts.

When botnets first started, they would frequently try to evade detection by authorities and government(s) by deliberately avoiding using or attacking their systems. But botnets are becoming smarter and smarter, and they can now recognize a wide range of detection methods. It is now possible to identify and steer clear of honeypots, which are intentionally made to be easy targets for botnets, to aid in preventing discovery [35,36]. With the introduction of Trojans and worms, the world of botnets saw a dramatic change in the early 2000s. Operating system flaws were exploited by worms like Code Red and Slammer, which propagated quickly, infecting a lot of computers and automatically attaching them to botnets without any human input. However, Trojans tricked users into unintentionally installing malicious software, increasing the scope and power of botnets [35].

The rise of so-called “zombie networks” peaked in the mid-2000s. A central command and control (C&C) server operated remotely over a network of infected machines [35,36]. Peer-to-peer connectivity and encryption are two further advanced tactics used by cybercriminals to evade cybersecurity professionals’ attempts to track down and take down these botnets. This period of ever-more-complex and evasive botnets was epitomized by the infamous Storm Worm, which first surfaced in 2007 [37]. Botnets have developed to target sensitive data, including login passwords and financial information, as the primary incentive for cybercrime has switched from simple mischief to financial gain. Banking Trojans such as Zeus and SpyEye proliferated and allowed attackers to commit enormous online banking frauds [38]. These botnets were customized for specific tasks, reflecting a more sophisticated and business-oriented approach by cybercriminals.

**Figure 3:**
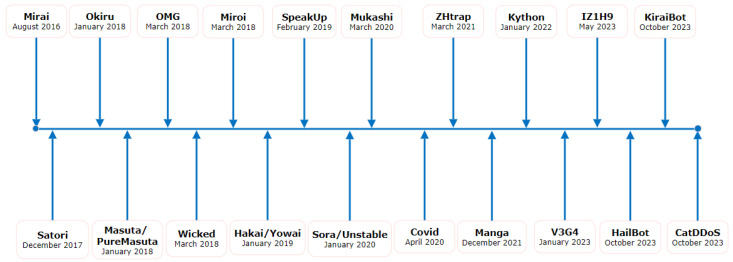
The evolution of Mirai botnet (2016–2023).

The Mirai botnet became infamous in 2016 when it used infected Internet of Things devices to carry out extraordinary DDoS operations and has evolved significantly over the years as shown in Figure 3. Mirai exposed the security flaws caused by the exponential increase in connected devices by making use of weak or default passwords in IoT devices [39]. This incident highlighted the importance of stronger IoT security measures and raised awareness about the risks of using vulnerable smart devices.

The Mirai botnet infected over 600,000 agents between August 2016 and February 2017, the majority of which were Internet of Things devices [39]. Since then, Mirai has already been linked to over 15,000 DDoS attacks. The source code for Mirai was originally made available to the general public on 30 September 2016. Numerous additional significant DDoS attacks have followed, including one that targeted the French web host OVH (1 Tbps) [40] and one that happened on 21 October 2016 [41] against Dyn, a DNS provider for popular websites like Twitter, Spotify, Netflix, Reddit, and GitHub. The most well known is the DDoS attack on writer Brian Krebs’ popular cybersecurity blog, which achieved a traffic volume of 623 Gbps—a level of data never before recorded or ever made public for a DDoS attack [42]. About a million users were impacted when a Mirai version in late 2016 took use of a flaw in the CPE WAN Management Protocol (CWMP) used in two models of Deutsche Telekom customer routers [43]. In 2017, Radware noticed that a botnet known as Brickerbot [44] started probing ports associated with the SSH service, specifically port 22. Furthermore, the Reaper variation was discovered [45,46]. It utilizes a portion of the Mirai code but concentrates primarily on attacking known vulnerabilities. The Reaper variation uses HTTP-based attacks of known vulnerabilities in the IoT devices instead of relying on Telnet brute force with default credentials [45]. A new Mirai variation called Satori surfaced in November 2017 [47]. Its unique spread mechanism makes it more worm-like than other variants. For remote planting, this bot does not rely on the loader–scanner method [48]. Satori asks compromised devices to download themselves from the same initial URL, targeting ports 37,215 and 52,869. Satori mostly exploits two vulnerabilities: one for port 52,869 that has been known since 2014 (CVE-2014-8361) [49], and another that was found in December 2017 (CVE-2017-17215) [50]. According to reports, the WICKED bot actively scanned ports 8080, 8443, 80, and 81 in 2018 [51]. After that, new exploits based on two vulnerabilities CVE2018-10561 and CVE2018-10562 related to the HTTP service authentication have begun to be included in at least five distinct botnet families [52].

Two vulnerabilities against GPON home routers were revealed by VPN Mentor on 1 May 2018 [53]. In 2018, the discovery of Okiru, a new strain of Mirai, focused on Internet of Things devices that have Argonaut RISC Core (ARC) CPUs. Similar to Mirai, the Okiru malware looks for devices using Telnet ports and attempts default passwords. The Masuta (Japanese for “master”) botnet appeared that year, and its source code was accessible on a secret invite-only dark forum. This botnet uses a different encryption key seed than Mirai and XORs the strings in the configuration files by 0 × 45 in order to take advantage of antiquated router flaws. Masuta’s improved version PureMasuta incorporates a list of vulnerable credentials that can be exploited and recycles popular Mirai-style malware. By taking advantage of a remote code execution vulnerability in the ThinkPHP framework, the Mirai variant began to spread in 2018 [54]. Due to this vulnerability, computers were forced to download and run malware, which then used Telnet to connect to other IP addresses. Yowai, which added the ThinkPHP vulnerability to the list of possible infection vectors, trailed Miori in 2019. Yowai is instructed to take over routers via port 6 in order to initiate DDoS attacks [55]. Another Mirai-based bot was identified in July 2019 called Moboot. It uses the same Mirai scanning mechanism to exploit many cooperating bots targeting DVRIP, ADB, HTTP, and Telnet-related ports [56,57]. Researchers discovered two variations in 2020, Sora and Unstable, using a novel propagation technique. Through CVE-2020-6756, these variations allow remote code execution on a certain video surveillance storage system. Unstable takes advantage of the previously disclosed vulnerability in ThinkPHP [58]. A version known as Mukashi first surfaced in 2020 and used a pre-authentication command injection vulnerability (CVE-2020-9054) to target network-attached storage (NAS) [59]. Figure 3 shows a comprehensive chronology with significant variations spanning from 2016 to 2023.

Botnets have been an essential part of sophisticated cyberattacks such as Advanced Persistent Threats (APTs) in recent years. Sophisticated, multi-purpose botnets are used by nation–state actors and well-funded cybercriminal groups for espionage, data exfiltration, and critical infrastructure disruption. With their high degree of adaptability and frequent use of sophisticated evasion strategies, these contemporary botnets are powerful opponents in the field of cybersecurity. IZ1H9, HailBot, KiraiBot, and CatDDOS are the most active Mirai variants as of 2023 [60].

## 6. Iot Botnet Detection

**Figure 4:**
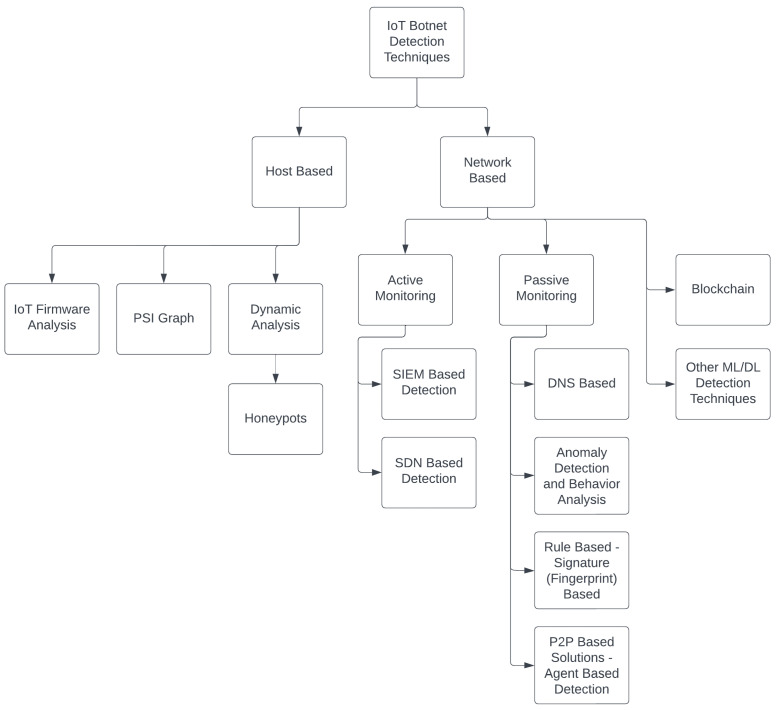
IoT botnet detection techniques taxonomy.

In the previous section, IoT botnets and their associated attacks are analyzed. This section focuses on IoT botnet detection techniques against explained attacks. Given the evaluative DDoS attacks posed by IoT botnets, IoT botnet detection techniques are a crucial step in preventing malicious activities of botnets within IoT devices and networks. This section analyzes the various detection techniques available for guarding IoT devices and networks. The proposed taxonomy of IoT botnet detection techniques is given in Figure 4. IoT botnet detection techniques are divided into two groups as host-based detection techniques and network-based detection techniques [61].

### 6.1. Host-Based Detection Techniques

Host-based botnet detection systems assess multiple aspects of a host’s behavior to find anomalies that might indicate a botnet infection. Table 3 summarizes host-based detection techniques and their details.

Host-based detection mainly focuses on the analysis of code on the device to detect botnets. These methods analyze processing time, access to unknown files, etc., to understand botnets. This type of detection system can be grouped into two distinct methods: static and dynamic analysis methods [71]. In the static method, both binaries and source codes are examined, while in dynamic analysis, devices are analyzed in real time.

Benson and Chandrasekaran [72] rang the bell to draw attention to the fragility of IoT systems. They focused on vulnerabilities that arise from not-botnet-infected IoT devices. They did not explain a botnet detection method, but they provided a valuable alert on the importance of host-based detection techniques.

As one of the static methods, Costin et al. [62,63] provide surveys in 2014 and then in 2018 on IoT firmware and detection techniques of malware in IoT Firmware. This is a significant source for analyzing firmware-related techniques. In these studies, they provided a way of analyzing firmware images to detect possible malware and botnets. Later, Nguyen et al. [64] propose another static analysis technique that analyzes the source code or binary executables of IoT firmware to find Printable String Information (PSI). Then, the PSI graphs are used for the Convolutional Neural Network (CNN) to train with malware samples. The PSI context is one of the most important pieces of information for obtaining better accuracy within the CNN classifier. A combined PSI-graph and CNN technique is used to detect other firmware to find out if IoT firmware is infected. Their evaluation results shows that the PSI-graph CNN classifier has an accuracy of 92%.

Zaddach et al. [65] propose a dynamic analysis approach that combines hardware (to analyze the input/output of an IoT system) and software to dynamically detect malicious firmware (botnet). Dynamic analysis is important in a security analysis of IoT systems, which allows dynamic taint tracing or symbolic execution. Zaddach et al. present a tool called Avatar which performs dynamic analysis to be used in vulnerability discovery, and detection. They provide a vulnerability analysis of the detection system to prove that their solution can be used to perform dynamic analysis of complex firmware.

After dynamic analysis techniques started to be applied for IoT botnet detection, IoT Honeypot-based solutions emerged to detect botnets. These honeypots act as targets to capture malware. Once IoT botnets attack these honeypots, the activity is recorded and appropriate mitigation strategies can then be applied. Pa et al. [66] provide the implementation of IoT honeypots. They propose IoTPOT to emulate Telnet services of various devices. This IoTPOT includes a virtual environment called IoTBOX to capture activities and analyze these activities. As a result of these analyses, they demonstrate a huge number of Telnet attacks and various botnet DDoS attacks on IoT devices. By this implementation and analysis, they detect at least five different botnet families, which shows the effectiveness of Honetpots. Because of these capabilities, there are various techniques that have been developed using honeypots. However, they have trouble detecting emerging IoT botnets, which are known zero-day attacks. With the development of machine learning (ML) solutions, honeypot data are also used to train ML models. Viskarma and Jain [67] propose a new detection technique using honeypots with ML algorithms. The IoT honeypot-generated data are used as a data source for the ML models. For the data collection, different types of IoT Honetpots are used, including IoTPOT [66], Dionaea, ZigBee Honeypot and other Multi-purpose IoT honeypots. These collected data are trained on different ML models such as CNN, RNN, and LSTM. They cannot use deep learning models due to a limited dataset. With these trained ML models, they are able to capture zero-day botnet types that are not trained in their model. This study does not include explicit experiments to prove their models, but they argue that this model has 99% accuracy rate, which shows the power of the hybrid model of honeypots with ML models. Banerjee et al. [68] also propose a similar ML-based honeynet solution. They collect malicious network traffic dump, binary files and log files using local honeypots. These collected data are used to train ML models. This trained ML model is tested and validated with the popular SocialNet dataset. Later, Memos and Psannis [69] propose AI-powered honeypots with the use of cloud computing. They create a novel honeynet that is composed of many isolated honeypots, and each of the honeypots operates as a decoy for the attacks. This honeynet is connected to a cloud server, where the analysis of attacks on the honeynets is conducted. The collected data in the cloud server train a supervised Logistic Regression model, which aims to predict infected hosts and networks. A trained model in a cloud server is used in real time to detect botnet existence. Once these models detect botnet, the cloud server can mitigate the attack in the corresponding IoT device. This strategy improves the accuracy rates of IoT botnet detection to nearly 100% in the authors’ experiments. This study demonstrates how hybrid techniques including honeypots, machine learning models and the cloud server can be effectively used for botnet detection.

Sajjad et al. [70] address another vulnerability of IoT devices within the Manufacturer Usage Description (MUD). Network access to IoT devices requires MUD to convey network-level functionalities. It is designed to increase the security of IoT devices on networks. However, Mirai botnets exploit the vulnerabilities of MUD. Hence, Sajjad et al. propose improvements to the MUD for IoT botnet prevention. These improvements suggest the generation of MUD profiles based on vulnerability scoring. The results of the study show that proposed changes improve the security level of services and IoT devices.

### 6.2. Network-Based Detection Techniques

Another approach to IoT botnet detection is through network-based detection techniques. Network-based botnet detection techniques involve monitoring and analyzing the traffic and patterns within IoT networks to detect botnet activities. This section delves into the network-based detection techniques. Network based detection techniques can be classified into two categories, active monitoring and passive monitoring [73].

The active monitoring technique probes the network proactively to measure the reactions of the network. It aims at identifying problems in real time. These problems include security threats and performance metrics. Active monitoring provides instantaneous insights but may increase the network load.

On the other hand, passive monitoring observes network traffic without interfering. This detection technique captures packets, analyzes logs, and finds anomalies and threats. This technique does not aim to prevent attacks in real time. It provides an in-depth knowledge of the past behavior of the network. Passive monitoring is used for post-analysis, future threat detection, and compliance reporting. Most of the detection techniques are a type of passive monitoring technique.

#### 6.2.1. SIEM-Based Detection Techniques

Major active monitoring detection techniques are grouped in SIEM (Security Information and Event Management) systems. Some major SIEM-based detection technique approaches are listed in Table 4. SIEM systems are primarily used in the security field to correlate events reported by various network security defense technologies (e.g., intrusion detection systems and firewalls) deployed within an enterprise network. The results of the correlation of events indicate the presence of a security incident.

In the paper [74], the authors propose a security solution solely based on security event management in the IoT domain which helps to detect malicious activities. The authors categorize different algorithms for generating the rules based on their characteristics. These algorithms will help in analyzing events, detecting anomalies, and correlating security-related information to detect potential botnet attack. They briefly discuss the attack scenarios on the confidentiality, integrity, and availability of IoT devices and describe the exploited vulnerabilities, the security events that are produced by the attack, and accurate defense responses that could be launched to help decrease the impact of the attack on IoT devices. The security events are particularly refined in the SIEM-based system model based on multiple relations between various categories of security events, attack surfaces, and vulnerabilities. The proposed multi-relations can help to investigate the event, as it also helps to identify the vulnerabilities that could have been exploited and the related attack surfaces inside the IoT devices. This proposed approach can be enhanced in the automatic generation of relations between the rules such that the SIEM system may be able to face various combinations of attacks, vulnerabilities, and events.

Basheer et al. [75] also focus on the SIEM solution-based detection technique, which is useful in detecting the IOT botnet DDoS attack. In the proposed architecture at the initial step, IoT traffic logs are forwarded by the default gateway to the SIEM system. These traffic logs are obtained from various IoT devices in the monitored network. The SIEM solution performs a sequence of data-processing tasks that include parsing, indexing, and storing these logs in a highly secured database. The logs are then analyzed, and if there is any abnormal behavior compared to the traffic profile of the device, it detects an attack and alerts the network administrator. The monitoring of various systems in real time could be a challenge for security analysts. With the use of Splunk, all relevant logs are collected and stored in one instance, which allows the designing of a single solution. The main aim of the authors proposing this prototype or architecture is to show that it is possible to detect different types of malicious traffic originating from various IoT devices. Marian et al. [76] also propose the use of the Splunk SIEM platform, which has been made to display four real-time alerts for the detection of various types of suspicious and/or malicious activity. One of the alerts is particularly designed for the identification of a Mirai virus infection within the company. They also propose the use of artificial intelligence combined with the SIEM to enhance the DDoS attack detection in systems. The utilization of artificial intelligence further enhances the detection capabilities of the system by enabling the system to learn and adapt to the ever-changing attack patterns, thereby improving the overall security of IoT environments.

#### 6.2.2. SDN-Based Detection

Software-defined networking (SDN) is a network management approach to control and manage the network dynamically using software applications [77]. SDN consists of data and control planes, which makes it different from traditional networks and enables the capability of programmable networks [78]. SDN requires reduced costs while offering a global view of the network. Due to these reasons, many detection techniques are developed based on SDN as summarized in Table 5.

Ozcelik et al. [79] propose edge-centric software-defined IoT defense (ECESID) architecture using the fog computing paradigm. This technique uses a threshold random walk with a credit-based rate limiting (TRW-CB) algorithm. This algorithm tries to detect the scanning phase of attacks on the host by relying on the likelihood of successful connection attempts. This mechanism exploits a queue of TCP SYNs for each IoT device to identify malicious activity.

There are techniques available that combine SDN with intrusion detection systems (IDSs). Manso et al. [80] propose a system which integrates the intrusion detection system (IDS) within the SDN architecture. This system includes three main components: the network, the IDS, and the SDN controller. This technique benefits the capability of IDS systems. IDS analyzes the incoming network traffic, finds malicious traffic, and sends an alert to the SDN for it to be handled. The SDN controller updates the network rules based on alerts coming from IDS. This approach ensures the fulfillment of three essential stages: detection, communication, and mitigation. This study shows how SDN can be used effectively with other techniques.

With the improvements in machine learning solutions, various detection techniques with ML have emerged that increase the capabilities of detection techniques in software-defined networks. Wani and Revathi [83] propose a technique that uses a combination of Naive Bayes and Principal Component Analysis (PCA) for the detection of Ransomware and DDoS attacks. In this method, the SDN controller extracts TCP/IP headers, which are then analyzed by ML algorithms to detect Ransomware and other attacks. This SDN-based solution provides detection and mitigation together to decrease threats to the IoT environment. Experiments in this study show that the proposed technique improves the accuracy of Ransomware and DDoS attack detection. Wani and Revathi [78] also suggest another method using Micro-Cluster Outlier Detection (MCOD), which includes Multi-layer Perceptron (MLP), to identify abnormal behaviors. In this study, the authors argue that most DDoS detection techniques are deployed directly on IoT networks which consume resources. Centralized SDN control can achieve better DDoS detection mechanisms in the IoT since it has enough resources to implement the necessary mechanisms. Based on this claim, this study proposes SDIoT-DDoS-DA, which is based on an SDN-based stateful solution for IoT devices. This proposed mechanism monitors the system, which detects anomalies. Then, Micro-Cluster Outlier Detection (MCOD) is used to decide whether the unusual behavior is a DDoS attack. This outlier detection uses multi-layer perception to detect DDoS attacks. As a result of this study, they prove that this technique can be used for DDoS detection and prevention due to the high accuracy and decreased resource consumption in IoT devices.

Ren et al. [81] design an effective detection mechanism using the genetic algorithm GA-XGBoost based on SDN. By using the OpenFlow protocol in SDN, it extracts six-dimensional vectors as input to the GA-XGBoost algorithm. The XGBoost algorithm is selected for this study because it has capability to solve the prediction and classification problems in limited processing capability controllers. This trained model is deployed on an edge controller with limited resources. This model is tested with collected data from the SDN network. As a result of the experiments, the detection rate of this model is found as 95.73%, and the false alarm rate is significantly lower than other ML algorithms within SDN. In another study, Wang et al. [82] utilize another machine learning algorithm, Dynamic Generative Self-Organizing Maps (DGSOMs). This study proposes a novel source-based detection technique using sFlow and Dynamic Generative Self-Organizing Maps (DGSOMs) for detecting DDoS attacks in SDN. This technique includes macro- and micro-detection. sFlow-based macro-detection covers the entire network to perceive DDoS attacks, and DGSOM is used as micro-detection to recognize the attack traffic. This micro-detection allows the system to differentiate the attack flow and the normal flow. There are also many other ML techniques used in SDN-based solutions as summarized in [25]. Refs. [84,85,86,87] apply detection techniques using Random Forest. In addition to those models, SVM [84,88] and KNN [84] are also widely utilized as machine learning techniques for classifying collected data in SDN-based detection applications.

Negera et al. [25] discuss that even if ML techniques show good performance, these techniques require extensive feature selection compared to deep learning models to achieve efficient attack detection. Hence, deep learning models for detecting attacks in software-defined networks have become much more prominent in recent studies. Assis et al. [89] suggest a Convolutional Neural Network (CNN) for the detection of DDoS for SDN sources. CNN is a DL model that is used for images; however, SDN IP flow traffic data are time-series data, not an image. They use a variation of CNN that is 1D-CNN. This proposed method is tested on different datasets. In the CicDDoS 2019 dataset, the CNN method achieves better results compared to MLP and Logistic Regression (LR) methods. Other studies [90,91,92] also imply CNN-based detection techniques in SDN. Recurrent Neural Network (RNN) and LSTM are two other deep learning techniques which are widely used in the detection of attacks in SDN-enabled IoT. Hasan et al. [93] implement an LSTM model integrated into SDN controllers. The model results in 99.96% accuracy in the state-of-art N_BaloT 2018 dataset. Alshraa et al. [94] and Malik et al. [95] implement RNN-LSTM models in SDN and test their models with different datasets. They show that LSTM requires more training time than RNN while having similar accuracy and false positive rates. All these studies demonstrate that different deep learning models can be used to detect DDoS attacks in SDN-based techniques. These models have higher accuracy than the ML models, but based on the requirements and data source size, different techniques can be used interchangeably.

#### 6.2.3. DNS-Based Detection

Previous detection techniques are a type of active monitoring that aims to detect in real time by adding additional network load. However, there are many detection techniques that involve passive monitoring [73] that analyze packets and identify anomalies. DNS-based detection techniques are one of the significant passive detection techniques. Different DNS-based detection approaches are shown in Table 6.

The DNS system is one of the most important elements of the Internet; it translates a domain name into an IP address, and vice versa. Quite notably, DNS helps Internet users locate various online resources, such as web servers and mail servers. Unfortunately, because of its basic functions, the DNS service is frequently involved in various malicious activities in one way or another.

Monika et al. [96] primarily focus on the various DNS-based detection techniques, such as anomaly-based traffic analysis at the ISP level using the EXPOSURE detection system, which operates at the ISP level and monitors the entire traffic for malicious domains. Deployment of machine learning at the local area network level using the BotGAD detection system uses machine learning techniques to identify malicious domains. They also discuss the Fast-Flux service network detection, in which they are using the FluXOR detection system for active probing techniques to detect abnormal domains and infected devices. The DGA-based detection named Pleiades operates at the enterprise or local area network level to discover the bots. The authors also propose that passive DNS analysis approaches such as an autonomous system is a group of one or more IP prefixes subdivided into groups, and the analysis of benign domains helps in the differentiation of benign domains from malicious domains based on a domain list from Alexa Top 500 by using DNS querying of each domain over 24 h. Through analysis of the FFSN domains, they can find out the benign domains from malicious domains by applying over the 10 ANS (Autonomous System Number).

Xingguo Li et al. [97] also propose DNS-based techniques like Fast-Flux (FF) and the Domain Generation Algorithm (DGA). In the Fast-Flux detection technique, the main focus is on identifying and tracking down the networks that rapidly change their IP addresses and proxies to hide the phishing websites and malware so that it is very hard to find the source server or the primary control server. They review these DNS detection techniques and suggest that there is a chance for advancement when considering large-scale networks where these algorithms might not work efficiently. The paper proposes strategies for mitigating the impact of botnets once detected. This may involve isolating infected devices, disrupting botnet command and control device, and implementing security measures to prevent future botnet infections. Xuan et al. [98] do not carry out the traditional techniques but use machine learning algorithms to detect the malicious bots on the DNS query data. The authors train the ML algorithms such as KNN, Random Forest, Decision Trees, and Naive Bayes using three datasets with 20,000 rows each and one test dataset with 20,000 rows. Here, the KNN algorithm gives accuracy results of 89.5%, 82.70%, and 94.10% which are similar to the Decision Trees accuracy results of 89.10%, 81.50%, and 93.40%, and the Random Forest algorithm gives the highest accuracy classification results for all datasets of 90.70%, 84.20%, and 94.40%, while Naive Bayes gives the lowest accuracy results of 83.10%, 82.80%, and 83.90%. Manmeet et al. [99] explain the evolution of DNS detection techniques for IoT botnets and classify them into five categories: flow-based detection, anomaly-based detection, flux-based detection, DGA-based detection, and bot infection detection techniques. The authors research these techniques and discuss the main attributes to consider in DNS datasets, such as real-time detection, versatility, scalability, and low false positives. A comparison is also performed based on the detection rate, FP rate, and FN rate for each category. This paper also compares each mentioned technique to determine which method works efficiently. The problem with machine learning techniques is the unavailability of a labeled real-world dataset for evaluation purposes, which is currently not available in large quantities. The dataset from a virtual setup does not completely resemble real-world data and is not suitable for real-time detection.

#### 6.2.4. Anomaly Detection and Behavior Analysis

Anomaly detection is the process of identifying anomalies or patterns in the network that do not conform to expected behavior. The key is to establish normal behavior patterns and identify behaviors that deviate from these patterns. Various anomaly-based protection techniques have been developed to effectively detect these deviations as shown in Table 7.

Borges et al. [100] propose an anomaly detection technique using a combination of multi-scale ordinal patterns transformation and Isolation Forest by first evaluating the number of packets a device transmits and transforming the constructed time series into a set of relevant features that represent the characteristics of the distinct dynamics of the devices’ operations. The transformation is applied to a given time series x of length m, using the embedding dimension and embedding delay parameters. The resulting features are then used as input for the Isolation Forest anomaly detection algorithm. By investigating how devices evolve, the solution can distinguish between normal and anomalous behaviors. Thus, Mirai and Bashlite, two major botnets for IoT, can be detected.

The paper [101] primarily focuses on the use of Traffic Flow Features as Metrics (TFFM) for detecting application layer-level DDoS attacks in IOT traffic flows. The TFFM approach uses three primary metrics to track the inflowing traffic: IP address, traffic growth rate, and similarity of traffic. These metrics are used to differentiate between attack-prone and benevolent traffic flows and to identify traffic flows that exhibit abnormal behavior.

Sudharsan et al. [102] address resource-constrained IoT devices (e.g., Microcontroller Units-based IoT), as they cannot perform huge computations. They propose an offline ML-based detection technique called Edge2Guard. They select an N-Balot dataset which includes pcap packet data. The attack traffic data are used, which include botnets from the Mirai and Bashlite families. These data are trained by Supervised Learning Models to capture anomalies in regular traffic. They achieve almost 100% detection rates with Random Forest and Decision Tree models. According to Sudharsan et al. this detection technique performs with the highest detection rates compared to existing approaches (both host-based and network-based models) in resource-constrained IoT devices.

Dytokinesis [108] is a novel anomaly detection technique that is inspired by the biological process of cytokinesis. It works by bisecting a dataset into normal and anomalous classes using Empirical Data Analysis (EDA) and Gaussian kernel. Dytokinesis is different from other anomaly detection techniques because it achieves significantly higher accuracy compared to other techniques as demonstrated by experimental results. Additionally, Dytokinesis has low latency and can work effectively on different types of IoT devices and networks.

Alzahrani et al. [103] propose a novel approach to identify network anomalies in the IoT using fog computing. The proposed solution combines three algorithms (KNN, EWMA, and CUSUM) to achieve high accuracy and a low false positive rate. This approach involves data pre-processing, feature selection, and categorization using machine learning models. The proposed mathematical model estimates the system’s quantitative behavior. The approach is evaluated in terms of experimental details, evaluation metrics, and experimental results and compared with other approaches.

Deep learning techniques, such as autoencoders and Deep Neural Networks, offer significant advantages over traditional methods in detecting IoT attacks and botnets. These advantages include the ability to detect emerging botnets, automatic feature extraction, flexibility to adapt to changing attack patterns, efficiency in processing large volumes of data, and proficiency in detecting anomalies. Overall, deep learning provides a more advanced and effective approach to IoT security by improving accuracy, adaptability, and efficiency in safeguarding IoT devices and networks.

The proposed approach in work [104] differs from previous techniques; it is anomaly detection using deep learning. In the first phase, an attack similar to a typical IoT botnet attack is simulated, which is referred to as the ‘unknown attack‘. The autoencoder is used to detect anomalies in the traffic generated by the unknown attack. The output of this phase is a set of detected anomalies. In the second phase, a multi-output Deep Neural Network (DNN) is used to classify the remaining detected known data into botnet and attack types.

Rambabu et al. [105] have also found out that the deep autoencoder could be more accurate than the Multi-Layer Perceptron (MLP) and Random Forest. Deep autoencoders are a type of artificial neural network that can learn to reconstruct input data, and they are commonly used for unsupervised learning tasks such as anomaly detection. What sets deep autoencoders apart from other anomaly detection techniques is their ability to learn complex patterns and features from raw data, making them well suited for detecting anomalies in large and diverse datasets generated by IoT devices. Deep autoencoders can capture intricate relationships within the data and identify deviations from normal behavior, leading to more accurate anomaly detection compared to traditional methods.

Similarly, Hairab et al. [106] propose an approach of using CNN and regularization techniques to help in detecting anomalies by reducing overfitting and providing a generalized model that can fit well on unknown data. At the same time, the regularized CNN model outperforms the standard CNN model, which does not use regularization, and this assists in improving the ability of CNN to identify anomalies in the IoT network. Additionally, Mahajan et al. [107] propose an autoencoder-based approach for detecting botnet attacks in IoT environments using unsupervised deep learning models. The method leverages the power of autoencoders to learn the underlying patterns and features of legitimate device behavior and identify potential botnet activities. The use of autoencoders allows the system to learn complex patterns, perform unsupervised learning, detect anomalies, and achieve high detection accuracy. Compared to traditional methods, deep learning offers advantages in adapting to evolving botnet attacks, utilizing unlabeled data, and providing superior performance in detecting botnet activities.

In conjunction with the detection method, an effective technique for localizing the anomalous data dimensions is also proposed. Mozaffari and Yilmaz [109] follow a nonparametric, i.e., data-driven, and semi-supervised approach, i.e., trains only on nominal data. The proposed technique is a sequential and multivariate anomaly detection method that scales well to high-dimensional datasets. The method applies to a wide range of applications and data types, and it can quickly and accurately detect challenging anomalies, such as changes in the correlation structure and stealth low-rate cyberattacks. The proposed method is evaluated using a real IoT-botnet dataset.

The proposed technique by Doshi et al. [110] is an anomaly-based intrusion detection system (IDS) called Online Discrepancy Test (ODIT) that can detect and mitigate stealthy DDoS attacks in IoT networks. The ODIT algorithm is based on statistical anomaly detection and is capable of detecting even very low attack sizes per source. The proposed IDS is computationally efficient, scalable to large networks, and does not rely on presumed baseline and attack patterns. The performance of the proposed IDS is evaluated using a testbed implementation, the N-BaIoT dataset, and simulations.

The Swarm Intelligence (SI) algorithm is a type of artificial intelligence that is characterized by self-learning, self-adaptation, and collective behavior to complete a particular task. The unique combination of self-learning, collective behavior, efficiency, adaptability, and superior performance sets Swarm Intelligence algorithms apart from traditional anomaly detection techniques and makes them well suited for detecting botnets in IoT networks. The paper of [111] discusses the use of the Improved Multi-Objective Particle Swarm Optimization (IMOPSO) algorithm, which showed better performance in detecting botnets in IoT compared to other algorithms.

Ahanger et al. [112] propose a novel technique for detecting botnet attacks in user-oriented IoT environments using a deep learning approach inspired by recurrent neural networks and a Bidirectional Long Short-Term Memory Recurrent Neural Network (BLRNN) in combination with efficient word embedding. The proposed technique uses a word embedding procedure to translate textual data into a tokenized integral format for use with the DL technique. The technique is assessed using numerous DL techniques and compared with state-of-the-art techniques based on a variety of attacks connected with the Mirai botnet. By leveraging DL, particularly BLRNN and word embedding, the paper showcases how increasing the data size can enhance statistical measures and improve the detection of botnet attacks in IoT environments. The bidirectional strategy employed in the DL model proves to be a superior technique over different data instances, highlighting the effectiveness of DL in enhancing botnet attack detection capabilities.

#### 6.2.5. Rule Based: Signature (Fingerprint) Based Detection

Signature-based detection identifies known malware, viruses, or network intrusions by matching data against a database of known patterns or ‘signatures’ [113]. Some signature-based detection techniques are listed in Table 8.

Neisse et al. proposed in 2017 an integrated approach to enhance the certification process of IoT devices using Model-Based Testing and policy-based management [114]. The approach includes security functional testing using Model-Based Testing (MBT) with TTCN3, model-based policy specification and enforcement using the SecKit toolkit, and post-certification monitoring to detect vulnerabilities and enforce policies dynamically. The goal is to detect vulnerabilities in IoT devices and introduce runtime policy enforcement capabilities to protect users from cyberattacks.

Kumar et al. [113] propose a network-based algorithm for detecting IoT devices infected by Mirai or similar malware. The algorithm uses Mirai traffic signatures and a two-dimensional subsampling approach to analyze packet traffic generated by the devices. The proposed algorithm is optimized to detect bots well before the actual attack, during the scanning phase itself. The performance of the algorithm is evaluated using a quantity called the average detection delay. The paper also discusses the deployment of the bot detection algorithm within a real-world network and suggests prospective actions that can be taken after the detection of bots.

Almseidin et al. [115] propose a detection approach for IoT botnet attacks using the interpolation reasoning method. The approach involves investigating network traffic to extract relevant network parameters, applying the resampling technique, checking for missing observations, searching for input parameters, eliminating other network parameters, and storing the top three input parameters for training and optimization. The approach uses the concept of the fuzzy system and performs the interpolation technique to reduce the size of fuzzy detection rules. The approach is designed and optimized using a real IoT botnet attacks dataset and considers the three groups of IoT botnet attacks (DoS group, Information gathering group, and information theft group).

Furthermore, a technique for optimizing firewall filtering in high-speed IoT networks by dynamically adjusting the order of firewall rules based on actively calculated statistics that adapt to traffic conditions in real time is proposed in [116]. The technique uses the concept of priority to prevent errors in filtering changes and considers the importance of a rule in a traffic match and its relevance to other rules. The system effectively reduces the number of packet matches while maintaining the same filtering effect, resulting in better firewall performance and reducing the chance of firewall overloading and crashing due to sudden massive traffic changes.

#### 6.2.6. P2P-Based Solutions: Agent-Based Detection

These methods leverage the principles of Peer-to-Peer (P2P) networks and agent-based systems to detect DDoS attacks. Agents are used to monitor IoT network traffic flows within their respective subnets. Agent-based systems leverage these autonomous software entities to enhance threat detection, incident response, and overall security posture. Some detection techniques are summarized in Table 9. Agents can gather data from various sources, analyze patterns and anomalies, and respond to security incidents in real time. By distributing security tasks among multiple agents, organizations can improve their ability to detect and respond to cyberthreats effectively [117].

Proposed in 2019, the agent-based system in [117] involves installing an agent in each IoT installation, such as a smart home, to monitor the network traffic of the devices. The agents are nodes of a complete undirected graph and can communicate with each other in a Peer-to-Peer (P2P) fashion. The main idea is to use agents to collect traffic metrics and then relay such information between them, without flooding the entire network. Effective detection of an ongoing DDoS attack is facilitated by the exchange of sufficient information among agents. The agents can utilize limited processing and memory resources, and a lightweight workflow is employed to ensure scalability. The agents to which infected IoT devices correspond can collaboratively detect an ongoing DDoS attack by summing up the observations each one makes for the devices attached to it. The main metric used for traffic measurement is the rate of packets moving in and out of the network.

The protocol in [120] uses lightweight agents installed at multiple IoT installations to detect DDoS attacks. These agents collaborate through exchanging traffic information; at the same time, they utilize a blockchain infrastructure to securely reach a consensus about the information metrics that are locally calculated at the gateways of the system. The blockchain smart contract ensures the integrity of both the procedure and the information.

Liang et al. [119] propose a detection technique using a multi-agent system. ‘Multi-agent’ commonly refers to either MAS (multi-agent systems) or MAT (multi-agent technology). MAS consists of numerous agents. These systems, through MAS, can be broken down into simpler, more manageable modules. Each agent in a MAS is responsible for specific tasks, mainly focusing on coordination and communication. These agents are entirely autonomous and can function independently or as part of a group within the MAS. Despite being developed in various programming languages and following different design patterns, these agents adhere to standardized communication methods, enabling inter-agent communication that is absent in single-agent systems. This paper proposes a hybrid intrusion detection system that uses machine learning techniques, anomaly-based middle agents, and specification-based components to detect and prevent attacks in IoT environments. The system also utilizes blockchain and multi-agent systems to enhance security.

Furthermore, in 2023, Abu Bakar et al. [118] proposed an intelligent agent-based detection system for DDoS attacks that uses machine learning algorithms to extract features from network traffic and classify normal and attack traffic. The system first pre-processes the network traffic data to remove noise and irrelevant information. Then, it trains different machine learning models on the pre-processed data to identify the most important features for detecting DDoS attacks. The best model is selected based on its accuracy in predicting network traffic. The selected features are then used to classify the network traffic into different types, such as normal, malicious, or suspicious. The system also incorporates traffic authentication mechanisms to enhance security. Deep learning techniques are highlighted as a significant advancement compared to traditional methods for detecting DDoS attacks. Traditional methods often rely on manual feature engineering and predefined rules to identify attacks, which can be limited in their ability to adapt to evolving attack strategies. Deep learning, on the other hand, offers the advantage of automatically learning features from raw data, allowing for more complex patterns and relationships to be captured. This can lead to improved detection accuracy and the ability to detect previously unseen attack patterns.

### 6.3. Blockchain-Based Solutions

Blockchain solutions are used for collaboration between multiple parties for botnet detection, which is not possible in centralized systems where every decision or identification of a botnet device has been made by a single system [121]. These techniques are mainly utilizes blockchain technology to increase effectiveness of other detection technique approaches as shown in the Table 10. In the paper [121], the authors propose a new blockchain technique to detect P2P botnets known as AutoBotCatcher, which considers that infected devices of the same botnet frequently communicate with each other and form groups. As such, the AutoBotCatcher is used to perform dynamic analysis on this group of IoT devices based on their network traffic flows to detect botnets. AutoBotCatcher uses a permission Byzantine Fault Tolerant (BFT) blockchain, which serves as a state transition machine that permits collaboration between pre-identified parties without any trust, which can be used to collect and audit the IoT devices network traffic flows to achieve the collaborative and dynamic botnet detection as blockchain transactions. In order to perform collaborative and dynamic botnet detection by collecting and auditing IoT devices network traffic flows as blockchain transactions.

Georgios et al. [120] propose a new lightweight blockchain solution that can be installed at each IOT device in order to detect DDoS attacks performed by these IOT devices. This technique will scan the outbound information of the device in order to identify possible victims of DDoS attacks. The contribution of this paper is a protocol that enables multiple agents that are installed on gateways of different sites of IoT installations, to collaborate on detecting DDoS attacks. These agents collaborate through exchanging traffic information, while, at the same time, they utilize this blockchain infrastructure in order to securely reach a consensus about the information metrics that are locally calculated at the gateways of the system. This paper also helps to identify the importance of proactive measures to combat the increasing threats of DDoS attacks using IoT botnets. By implementing the collective intelligence of IoT devices through lightweight agents and blockchain technology, organizations can enhance their capabilities to defend against DDoS attacks.

Shafi et al. [122] introduce an innovative solution by combining the SDN with the distributed blockchain technique. The authors explain the changes in the architecture flow that can combine the distributed blockchain with the SDN technique. It can quickly download flow rules across the SDN controller blockchain network, look for modification or unusual behavior or traffic destined for a specific destination, and detect the DDoS botnets developed. It can identify DDoS botnets and traffic towards specific destinations. It can detect changes made to the system data, any topological features modification, and flow mode communication status to recognize malicious updates. This detection system is fully automatic, so no one needs to be involved manually.

With the emergence of blockchain technology using multiple platforms like Ethereum, it has become advantageous to focus more on blockchain solutions. Many of the blockchain IoT detection techniques are being built using the Ethereum platform. In this paper [123], a Blockchain Edge computing Hybrid System (BEHS) is implemented to make use of blockchain along with edge computing and provide secure IoT services. To secure data privacy and authenticity, a data access control scheme is designed by integrating symmetric encryption with an asymmetric encryption algorithm. The paper implements a concrete BEHS on Ethereum and the function of the PoC mechanism using smart contracts, and conducts a case study for a smart city. The evaluations and analyses show that the proposed PoC mechanism can effectively detect and automatically manage the behavior of nodes; the cost of the data access control scheme is within a reasonable range, and there is a chance for improvement in concurrency delays caused by smart contracts and a limited range of sensing devices. This paper [124] also uses Ethereum in their proposed blockchain technique to detect and prevent DDoS attacks against IoT systems. The proposed system will help to guard the IoT devices by helping to resolve issues related to single points of failure, privacy, and security. The proposed system uses a decentralized platform to prevent attacks at the application layer by authenticating and verifying these devices. The tracing and recording of IP addresses of malicious or infected devices is implemented using blockchain, which helps to isolate them, preventing them from connecting to IoT device networks. The evaluation helps to determine the advantage of the system because fewer I/O operations occur in the proposed system compared to other related works, making this system substantially faster.

In [125], the authors propose a safe digital framework that uses Blockchain technology that helps in the early detection of the formation of botnets in a smart factory environment. To collect data and inspect network packet headers from various devices using deep learning for connections with the external unique IP addresses and open connections, a collection of devices in the edge layer is developed to create a Digital Twin (DT). The data transmission from the corrupt devices is detected by synchronizing the data between the Digital Twin (DT) and a Packet Auditor (PA). The DT and PA are authenticated using the smart contracts, which ensures that the malicious nodes do not participate in the data synchronization, and botnet spread is prevented using the DT certificate revocation.

In this modern world, every person uses a lot of IoT devices for their everyday tasks, and some of these devices collect information for government work. In a smart city, Internet of Things security is essential. IoT security is a serious concern due to the many objectives and various drawbacks that can prevent the quick acceptance of these devices. The permission-based blockchain system proposed in this paper [126] employs lightweight technology and the arbiter PUF architecture to secure key pairs of Internet of Things devices. Because the machine learning-based ensemble technique has a lower false-positive rate and a higher detection rate than the other classification technique, it is initially employed in a collaborative detection system to identify DDoS attacks on Internet of Things devices. Subsequently, the authors in this paper [126] integrate blockchain technology, which securely sends warning signals to every IoT network node with sufficiently secure authentication.

There are significant research studies on the combination of blockchain with intrusion detection systems. The authors of the paper [127] propose to develop an intrusion detection system using machine learning and blockchain. This paper proposed a machine blockchain framework (MBF) to provide a distributed intrusion detection system with security and use the blockchain with the help of smart contracts in IoT device networks. This paper also demonstrates that the machine learning models, such as the Random Forest algorithm and proposed XGBoost algorithm can accurately detect malware in certain traffic instances. The XGBoost algorithm is designed to work with sequential network data, and the intrusion detection approach is trained using the N-BaIoT dataset. The data from the IoT botnets can be considered a dataset and can help train these machine learning models, which helps to safeguard the IoT device network from future malware. The data from three different devices, Provision_PT_737E Security Camera device, Philips_B120N10 Baby Monitor, and SamsungSNH1011N Webcam devices, are used to check the performance of the XGBoost algorithm with a comparison of Logistic Regression (LR), Random Forest (RF) algorithms. The data from the devices are severely unbalanced and normalized using z-scores as part of the pre-processing. The normalized data are used to train and test the three algorithms for accuracy in detecting the IoT botnets. The proposed algorithm XGBoost gives high accuracy results for three devices with 97%, 98%, and 98%, whereas the RF algorithm gives the accuracy results of 92%, 94%, and 94%, and the LR algorithm gives the accuracy results of 85%, 86%, and 83% which clearly shows that the proposed algorithm XGBoost has more accurate results.

### 6.4. Other ML/DL-Based Solutions

In previous sections, we analyze host-based, network-based, and blockchain-based detection techniques. Some ML/DL methodologies are combined with these techniques to enhance the performance. However, there are additional ML/DL methods that are combined with different detection techniques. In this section, we focus on explaining these additional methods.

#### 6.4.1. Machine Learning

Machine Learning based techniques are developed to enhance the detection performance of other techniques identifying patterns, and detecting anomalies. Some major ML-based detection techniques are summarized in Table 11.

The paper from Nanthiya et al. [128] utilizes machine learning algorithms, including Support Vector Machine (SVM), Decision Tree, and Random Forest, to detect DDoS attacks in IoT using the IoT-23 Botnet Dataset. Additionally, Principal Component Analysis (PCA) is employed as a dimensionality reduction technique to enhance the performance of algorithms. The study compares the efficiency of PCA with and without PCA results, evaluating algorithms using parameters such as accuracy, precision, F1 score, and recall. The results indicate that PCA significantly reduces the execution time while yielding similar results to those without PCA. Furthermore, the Decision Tree and Random Forest algorithms are found to accurately classify DDoS packets compared to SVM. The models in the study are trained on the pre-processed IoT-23 Botnet Dataset using machine learning algorithms such as SVM, Decision Tree, and Random Forest. Then, they are validated by testing on separate datasets to ensure accurate predictions. Adjustments are made based on validation results to optimize the models for real-world data.

In addition, Aysa et al. [130] employs feature extraction to gather 115 features from client gadgets, followed by feature selection to identify a subset of 40 key features using the Pearson coefficient technique. This research mentioned the use of standard datasets for two well-known DDoS attacks, namely, Mirai and BASHLITE. These datasets are collected before and after the infection of different IoT devices and are structured in CSV format to overcome data variety challenges. Using various machine learning and data mining algorithms such as LSVM, Neural Network, Decision Tree, and Random Forest, LSVM utilizes various machine learning and data mining algorithms to detect abnormal activities, including DDoS features. The experimental evaluation demonstrates that the merge between the Random Forest and the Decision Tree achieves high accuracy in detecting attacks. Collectively, these techniques form the basis of the proposed framework for IoT DDoS attack detection using machine learning.

Furthermore, the research article [129] uses machine learning techniques to detect botnet attacks in Internet of Things (IoT) devices over a cloud environment. The authors evaluate the performance of various classifiers such as Artificial Neural Network (ANN), Support Vector Machine (SVM), Decision Tree (DT), Random Forest (RF), K-Nearest Neighbor (KNN), Gradient Boosting (GB), and others. They also discuss the importance of feature selection for malware classification and intrusion detection. The authors use the Knowledge Discovery and Database (KDD) dataset and the N-BaIoT dataset, consisting of benign and malicious records for testing on each IoT device, which consists of five million samples of captured packets in the network, to evaluate the classifiers. The training process involves using a portion of the dataset to train machine learning models, while the validation process assesses the performance of the models in a separate portion of the dataset to ensure that they generalize well to unseen data. The study shows that the Passive Aggressive classifier achieves up to 98.4% precision score on binary classification, while DT regression attained an 89.5% precision score in multi-class classification.

As a further advancement, Malik et al. propose a solution with one-class KNN [131] as the primary one-class classifier, which has shown the best performance among one-class classifiers, achieving an F1-score of 98% to 99% on different IoT datasets. The model in the paper is trained on real-world IoT datasets collected from a consumer IoT gadget network, include traffic generated by three types of IoT botnets, Mirai, Bash lite, and Torii, capturing normal and malware traffic. Feature selection methods are used to reduce the feature space and select important features impacting performance. At the same time, the development of an efficient feature selection mechanism renders the proposed technique a lightweight solution for IoT devices, aiming to reduce the computational overhead and achieve a satisfactory detection rate with low false alarm rates.

The paper of [132] employs ensemble learning techniques, specifically Gradient Boosting Decision Trees (GBDT) and Random Forest, to detect and prevent IoT botnet attacks. The models in the paper are trained using the entire N BaIoT dataset, which contains a large volume of instances related to IoT devices.These ensemble methods combine multiple weak learners to create a strong model for the accurate identification of potential threats. Additionally, feature selection is utilized to identify the most prominent features for modeling training, enhancing the accuracy of the detection system.

#### 6.4.2. Deep Learning

ML-based detection techniques are effective methods. However, deep learning approaches are more widely used methods for the detection of DDoS attacks in IoT. Some common approaches are listed in Table 12. The paper [133] uses the CNN model trained using a dataset containing benign and DDoS attack packets. To validate the model, various validation methods such as cross-validation, subsampling, and repeated cross-validation are employed on novel labeled datasets. Grid search algorithms are utilized to identify the most effective learning features of the CNN for each dataset. This validation process helps ensure the accuracy and reliability of the model in detecting DDoS attacks in IoT networks. It achieves a high accuracy rate of 99.98% in classifying benign traffic and DDoS attacks. The methodology involves the collection of relevant datasets, the extraction of features specific to DDoS attacks, and the implementation of the CNN model for accurate detection. The dataset used in the paper consists of 95,000 benign packets and 125,000 DDoS attack packets collected from various sources. These packets are stored in pcap files and are analyzed for prediction and classification purposes. Additionally, the paper discusses the importance of mitigating real-time IoT DDoS attacks by capturing flood traffic in the network and applying Deep Neural Network techniques for prevention.

In [135], the authors discuss the bidirectional long short-term recurrent neural network, feed-forward neural network, and malware image classification. They also suggest a four-step solution for mitigating future DDoS attacks and adapting to current attacks. In one approach, the authors split the attack type into training and validation, with each model trained over twenty iterations. Another approach involves training a deep learning model on the UNSW-NB 15 dataset, with tenfold cross-validation on the entire dataset. The authors suggest adapting current attack patterns using machine learning to recognize attacks from specific locations, repeating offending IP blocks, or the improper use of particular protocols to strengthen the protection system for future attacks. The proposed solutions aim to assess DDoS attack detection in a setting more connected to the real world.

The paper in [136] leverages dimensionality reduction techniques such as PCA and autoencoder to reduce feature dimensionality, making it feasible to use deep learning algorithms like LSTM and CNN for botnet attack identification. The model is trained using deep learning algorithms such as LSTM and CNN. LSTM, a type of artificial recurrent neural network, is utilized for sequence modeling, while CNN is used for feature extraction from the input data. The researchers implement a lightweight detection system using a combination of PCA, CNN, and LSTM algorithms. Additionally, the study explores the use of unsupervised algorithms for future enhancements in botnet attack detection. The model in the paper is trained, validated, and adjusted using the Bot-IoT dataset, which is a publicly available dataset containing information about botnet attacks, regular traffic flows, and various cyberattacks in IoT networks. The training process involves pre-processing the dataset by removing unnecessary information, handling missing values, and encoding labels. Dimensionality reduction techniques like PCA and autoencoder are applied to transform the dataset into a suitable format for machine learning purposes.

Roopak et al. [134] propose and evaluate four different deep learning models for the detection of DDoS attacks in IoT networks: MLP (Multi-layer Perceptron), 1d-CNN (Convolutional Neural Network), LSTM (Long Short-Term Memory), and CNN+LSTM (hybrid model). The models are compared with traditional machine learning algorithms such as SVM, Bayes, and Random Forest. The performance of the models is measured using standard metrics such as accuracy, recall, and precision. The results show that the CNN+LSTM model performs the best with an accuracy of 97.16%, outperforming both the other deep learning models and the traditional machine learning algorithms.

## 7. Emerging Attacks and Detection Systems

In the previous sections, we explain detection techniques and IoT DDoS Botnet attacks that have occurred in the past. As the use of IoT devices continues to expand, the threat landscape is constantly increasing [60]. In this section, we explore the emerging threat of IoT botnet DDoS attacks and techniques developed to detect them.

### 7.1. Emerging IoT Botnet DDoS Attacks

Attacks using botnets are increasing significantly each year with a strong impact on different areas such as finance, entertainment, and telecom. The recent trends show that the attackers are increasing their attention on government, healthcare, and transportation systems. According to the NSFOCUS Global DDoS Attack Landscape Report, the DDoS attacks have been increasing steadily over the past 4 years [60], and the DDoS attacks report by StormWall states that 2023 has witnessed 68% year-over-year increase in DDoS attack [137]. One of the largest DDoS attacks was observed in April 2023 on a cryptocurrency platform, where the attackers unleashed a record-breaking 15.3 million requests per second. Cloudflare experts identified approximately 6000 botnets responsible for the attack, which were capable of making up to 10 million requests per second, and they originated from 112 different countries [138].

Some of the biggest attacks have been made in the last couple of years in every sector, leaving a large and long impact on society. Global events like the Russia–Ukraine war and NATO bids have driven the recent attacks. The Ministry of Defence and the Armed Forces of Ukraine were hit by a DDoS attack in February 2022 [60]. The US airports’ sites were taken down by pro-Russian hackers in DDoS attacks in October 2022 [60]. Russian hacktivists took down Norway government sites in DDoS attacks in June 2022 [60]. The impact of attacks is directly related to the sector of society. Attacks in sectors such as health care and energy have the potential to cause significant damage; unfortunately, the frequency of these attacks in these sectors is increasing every day. Beijing’s health code app called Jiankangbao, suffered a cyberassault from distant places on April 28. In July 2022, the Lithuanian energy business Ignitis Group experienced a cyberattack that it referred to as its “biggest cyberattack in a decade” due to multiple distributed denial of service (DDoS) strikes that caused disruptions to its websites and digital services [60]. Even private institutions like the Nobel Foundation were the victims of the DDoS attack on the award day in December 2021 [60].

**Figure 5:**
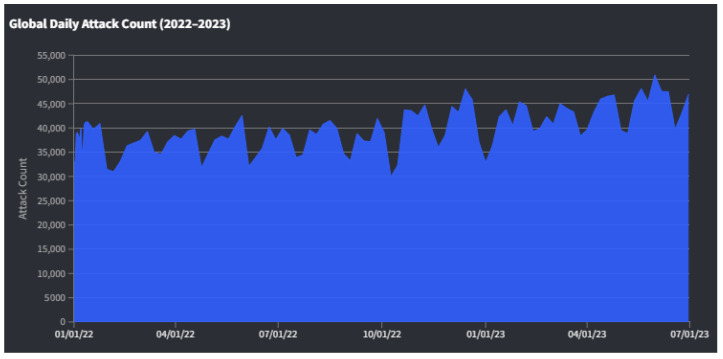
Global Daily Attack Count in 2022–2023. (Source: Netscout Threat Report 2023 [60]).

IoT botnets are used in most of the attacks that were mentioned above. According to Netscout’s 2023 report, experts in Netscout have identified 592,373 active botnets (until July 2023) across 235 countries and territories, and approximately 559,693 bots were involved in the targeting of enterprises [60]. Figure 5 shows the global daily number of attacks in the year 2022–2023 (until July 2023). As observed in the graph, the number of attacks has steadily increased over the year, indicating the need for greater caution.

### 7.2. Emerging IoT Botnet DDoS Detection Systems

In detection systems, there has been a lot of development from traditional methods to state-of-the-art approaches like ML [139,140,141,142], blockchain [143,144], AI [145,146,147], and DL [148,149,150]. In the early stages, traditional methods have relied heavily on signature-based detection, DNS, and SIEM to identify and prevent security threats. As IOT botnets evolved, these methods proved incapable, as they struggled to keep up with constantly evolving IOT botnet attacks. With the implementation of ML, AI, blockchain, and DL techniques, there has been a significant improvement in IoT botnet attack detection. The huge data produced by the devices can be analyzed by using ML algorithms, which helps to detect unknown threats/malicious approaches. Behavior analysis has become crucial to understanding the dynamic nature of IoT devices and helping to identify anomalies. Also, the integration of threat intelligence and collaborative sharing platforms have helped to increase detection capabilities with the usage of a collective knowledge base. Cloud-based solutions [151,152] were developed, and with this technique, it has become possible to analyze the data from different devices centrally at one location. At present, advanced techniques such as DL, AI, and ML are used to increase the precision and accuracy of IoT botnet detection. These models can learn automatically by using the data and adapting to new threats, which makes them more effective in identifying various old and new attacks. The collaboration among cybersecurity professionals, companies, and researchers has helped in producing new detection techniques that are helping to detect IoT botnet threats. There is a need for the continuous improvement of detection techniques, which ensures a trustworthy detection technique against the evolving IoT botnet threats. However, the primary challenge remains in consistently innovating or enhancing existing detection methods to counter evolving attack strategies, which is essential for securing the devices from new threats and ensuring the scalability and effectiveness of detection techniques in the everyday emerging domain of IoT devices.

## 8. Discussion

In this study, numerous studies in the literature on IoT botnet DDoS attacks and detection techniques are analyzed, and a comprehensive review is provided. This study aimed to cover various emerging types of DDoS attacks and state-of-the-art detection techniques through a detailed analysis of the literature.

After conducting a comprehensive analysis of the existing literature, it has become evident that there is an alarming trend in the realm of Internet of Things (IoT) devices. Despite considerable technological developments, these electronic devices still have inherent security risks which should not be ignored. These vulnerabilities are primarily caused by limitations in the cost, size, and computational capabilities of these devices. As a result, IoT devices are among the top targets for security breaches, and malicious malware can easily compromise them. Once compromised, attackers can use these devices as part of IoT botnets that can carry out large-scale attacks such as Distributed Denial of Service (DDoS) attacks. These attacks can cause significant disruptions and also pose a threat to the security and privacy of individuals and organizations.

On top of that, there is an ongoing evolution of IoT botnet attacks, emphasizing the importance of continuous research and analysis to address emerging threats. This paper examined how attackers constantly adapt their techniques, such as exploiting new vulnerabilities in IoT devices, to conduct DDoS attacks. The emergence of powerful botnets such as Mirai has led to large-scale DDoS attacks in various sectors, highlighting the need for proactive measures to strengthen the security resilience of IoT devices and the urgent need for robust detection techniques.

As shown in the paper, various robust detection techniques are available in the literature. Although traditional detection techniques, such as signature-based detection, have failed to keep up with increasing threats, breakthroughs in machine learning (ML), Artificial Intelligence (AI), blockchain, and deep learning (DL) provide the potential to improve detection capabilities. Those approaches are often combined with traditional detection techniques and provide high detection rates. These cutting-edge techniques create new best security practices, especially in protecting against DDoS attacks originating from botnets. However, the evolution of IoT botnet attacks requires the continuous development of cutting-edge techniques in this sector. As explained in the Section 9, there are still many open questions and future work available in the studies. This creates research opportunities in the corresponding fields.

## 9. Open Questions and Future Work

This study on the detection techniques of IoT botnets displays that researchers are actively working on new techniques to detect IoT botnets and minimize their impact. However, the study also shows that new attacks have been found frequently, indicating that there are factors that need to be considered in future research. This section lists a few open questions from analyzed techniques that are to be addressed and considered in the future research:

- Investigating low-rate spoofing DDoS or other attacks on SDN traffic with DL offers a complex research environment. Nadeem et al. [24] propose botnet detection in SDN-enabled IoT using deep learning (DL) techniques. However, identifying relevant characteristics in SDN traffic for efficient DL-based threat detection is still an important question. Researchers need to reconcile anomaly and signature-based detection while investigating the generalizability and flexibility of DL models across various SDN systems. Significant factors include maximizing resource efficiency and comprehending how resilient DL models are against adversarial attacks. Dynamic network adaptability remains a key open question in advancing the field.
- Antonia et al. [20] analyze the evolution of Mirai Botnet. Their study shows that Mirai signature is still extensively implemented by attackers. Their study concludes by possible methods of reducing hijacked devices with investigation of Mirai botnet signatures. How can network operators effectively reduce the occurrence of compromised IoT devices by analyzing Mirai signatures derived from investigations of Mirai botnet scans? What features of Mirai signatures specifically may be used as reliable indications for locating infected devices within a network and mitigating their impact? How can the integration of machine learning and anomaly detection techniques augment the effectiveness of signature-based approaches in identifying Mirai-infected IoT devices, especially in the context of emerging sophisticated attack patterns? Future studies on these questions can help researchers to understand the behavior of Mirai botnets better and give an opportunity to find a way to reduce the occurrence of hijacked devices.
- Pynadath et al. [104] propose multi-phase anomaly detection using deep learning. Their models can achieve high accuracy in detecting unknown IoT attacks and also classifies known data into their respective categories. They mention that this model can be used within network intrusion detection systems to detect all kind of IoT botnet attacks. In order to achieve this, the following questions should be considered to apply these models into other attacks. How can the application of anomaly detection techniques, specifically leveraging autoencoders and multi-output DNN, be effectively integrated into NIDS? How can multi-output DNN architectures be structured to comprehensively identify diverse anomalies across different attack vectors, ensuring a robust defense mechanism? Furthermore, what steps may be taken to maximize these methods’ scalability, efficiency, and interpretability while reducing computational overhead to meet the needs of large-scale, real-world network environments?
- The paper of Borges et al. [100] proposed an approach of Isolation Forest for anomaly detection. This method investigates how devices evolve and then distinguishes between normal and anomalous behaviors. But this model is only tested on Mirai and Bashlite botnets. Is it possible to use transfer learning and online strategies to follow the dynamical evolution of the botnets to detect other botnets with a proposed model? Further research on this research question can allow researchers to extend this methodology for all kinds IoT botnet attacks and other possible attacks.
- The paper of Shao and Chao [116] demonstrates a novel approach to firewall filtering in high-speed IoT networks by dynamically adjusting the order of firewall rules based on actively calculated statistics that adapt to traffic conditions in real time. How can this technique be optimized for more firewalls in networks that create excessive CPU use? Even if the proposed approach demonstrates an effective approach to reducing the number of packet matches while maintaining the same filtering effect, the same type of traffic still grows, which causes CPU overload. If this problem can be solved, this approach can be an effective and efficient way of detecting IoT DDoS attacks.

In this paper, different detection techniques are covered, which use different datasets for their validations. Therefore, it is not possible to compare their efficiency and accuracy against each other. The future work on the literature review of IoT botnet detection techniques needs to focus on the validation and comparison of the detection techniques through the integration of external datasets from different network environment. This integration can enhance the applicability and accuracy of available techniques under various circumstances.

## 10. Conclusions

Threat Intelligence Report 2023 [153] by Nokia noted that DDoS attacks using IoT bots have jumped five times in 12 months. The first finding of this report reveals that more than 60% of mobile network attacks are related to IoT botnets, although it gets worse every year. This shows the importance of research conducted in this field. IoT botnets have been evolving since they first emerged, and detection techniques need to evolve at the same pace. Therefore, considerable research is being conducted in this field. In this study, we have presented a comprehensive systematic review of the literature of those studies on IoT botnets in terms of attacks, state-of-the-art detection techniques, and current trends.

This paper contributes to the literature by providing an up-to-date comprehensive analysis of IoT botnet DDoS attacks, a systematic analysis of detection techniques, and a systematic taxonomy of these techniques. This paper aimed to deliver a comprehensive systematic literature review with IoT botnet attacks and detection techniques encompassing the recent research and future research opportunities.

This study first demonstrates IoT botnets, attack architectures, and the evaluation of IoT botnets, which shows that this thread is becoming more common and worse each year. Then, it lists the main techniques used to detect those attacks by identifying their key features. This paper also provides various detection techniques including ML/DL solutions which are improved to the current detection methodologies. These detection methods are organized into a systematic taxonomy to highlight their essential characteristics. Our goal is for this taxonomy to assist future studies in this domain. To encapsulate the latest research in this domain, current threads and recent detection techniques are explicitly discussed.

Despite all the research conducted in this field, many challenges remain in this area. We aim to present this review of the literature to assist future research in related fields. By providing current threads and detection techniques, the information is provided as a source for new studies to be conducted in this field. To support future studies, unresolved open questions are discussed in the Section 9.

## Figures and Tables

**Table 1:** Research focus areas of literature reviews conducted.

Year	Paper	Number of Times Cited	Focus on IoT Domain	Focus on Botnet and Types	Attack Architecture and Types	Evaluation of Attacks	Focus on Botnet DDOS Attacks	Analyze Different Detection Techniques	Taxonomy of Botnet Attacks and Detection Techniques	ML/DL Solutions	Current Threats and Trends	Open Questions and Discussion
2009	A survey of botnet and botnet detection [5]	192	✓	✓	✗	✗	✗	✓	✗	✗	✗	✗
2013	Botnets: A survey [6]	310	✓	✓	✓	✓	✗	✓	✓	✗	✗	✗
2015	A survey on Botnet: Classification, detection and defense [7]	13	✓	✓	✓	✗	✗	✗	✗	✗	✗	✗
2017	A survey of distributed denial-of-service attack, prevention, and mitigation techniques [8]	200	✗	✗	✓	✓	✗	✓	✗	✗	✓	✓
2020	A survey of DDoS attacking techniques and defence mechanisms in the IoT network [9]	149	✓	✗	✓	✓	✓	✓	✓	✓	✗	✓
2020	Survey on Artificial Intelligence Based Resilient Recovery of Botnet Attack [10]	2	✓	✓	✓	✗	✗	✓	✗	✓	✗	✗
2020	Distributed denial of service attacks and its defenses in IoT: a survey [11]	95	✓	✗	✓	✓	✗	✓	✗	✓	✓	✗
2021	Detecting Internet of Things Bots: A Comparative Study [12]	9	✓	✓	✓	✓	✗	✓	✓	✓	✓	✓
2021	Survey on botnets: Incentives, evolution, detection and current trends [13]	19	✓	✓	✓	✓	✗	✓	✓	✓	✗	✗
2021	IoT-based botnet attacks systematic mapping study of literature [14]	8	✓	✓	✓	✗	✗	✗	✗	✗	✗	✗
2021	Detection of Distributed Denial of Service Attack in an Internet of Things Environment—A Review [15]	6	✓	✗	✓	✗	✗	✓	✓	✓	✗	✗
2021	Internet of Things Applications, Security Challenges, Attacks, Intrusion Detection, and Future Visions: A Systematic Review [16]	101	✓	✗	✓	✓	✗	✓	✓	✓	✓	✗
2022	Deep learning approaches for detecting DDoS attacks: a systematic review [17]	18	✗	✗	✗	✗	✗	✗	✗	✓	✗	✓
2022	Blockchain Based Solutions to Mitigate Distributed Denial of Service (DDoS) Attacks in the Internet of Things (IoT): A Survey [18]	27	✓	✗	✓	✗	✗	✗	✓	✗	✓	✓
2022	A Taxonomy for Internet of Things in Security Distributed Denial of Service Attacks [19]	0	✓	✗	✓	✓	✗	✓	✗	✓	✗	✓
2023	The evolution of Mirai botnet scans over a six-year period [20]	0	✓	✓	✓	✓	✗	✗	✗	✓	✗	✗
2024	This Paper	-	✓	✓	✓	✓	✓	✓	✓	✓	✓	✓

**Table 2:** Paper selection steps for literature review strategy.

Step	What Is Performed in This Step	Number of Papers
Initial DTUFindIt Query	(IOT OR “Internet of Things”) AND Botnet AND security	1125
Extended DTUFindIt Query	(IOT OR “Internet of Things”) AND Botnet AND (DDOS OR Denial-of-service) AND Detection AND Attack	328
Initial Exclusion	With initial/exclusion criteria	300
Title/Abstract Analysis	Include botnet DDoS attacks or detection techniques	144
Snowballing Strategy	Additional papers + literature review papers from different queries	183
Full Paper Analysis	Individual full paper analysis based on research questions	102

**Table 3:** Host-based detection approaches.

Year(s)	Paper(s)	Detection Technique Features
2014, 2018	[62,63]	Analysis of IOT Firmwares
2018	[64]	PSI graph to feed in ML
2014	[65]	Dynamic Analysis
2016, 2019, 2020	[66,67,68,69]	IOT Honeypots
2020	[70]	Manufacturer Usage Description (MUD) improvements

**Table 4:** SIEM-based detection approaches.

Year(s)	Paper(s)	Detection Technique Features
2018	[74]	Event-Based Approach Using SIEM
2020	[75]	SIEM-based detection and mitigation
2021	[76]	Integration of Splunk Enterprise with SIEM

**Table 5:** SDN-based detection techniques.

Year(s)	Paper(s)	Detection Technique Features
2017	[79]	SDN-Based IoT Defense using Fog Computing
2019	[80]	Intrusion Detection Systems (IDS) within the SDN Architecture
2020	[78]	Micro-Cluster Outlier Detection (MCOD) within SDN
2022	[81]	SDN network and the OpenFlow protocol with XGBoost detection algorithm
2022	[82]	sFlow collected traffic statistics using clustering algorithm DGSOM
2022	[25]	Botnet Detection in SDN-Enabled IoT Using Machine Learning (ML) Techniques
2023	[24]	Botnet Detection in SDN-Enabled IoT Using Deep Learning (DL) Techniques

**Table 6:** DNS-based detection approaches.

Year(s)	Paper(s)	Detection Technique Features
2017	[96,97]	Analysis of DNS-based detection technique
2018	[98]	ML techniques using DNS Query Data
2019	[99]	Issues and challenges in DNS-based botnet detection

**Table 7:** Anomaly-based detection approaches.

Year(s)	Paper(s)	Detection Technique Features
2023	[100]	Multi-scale ordinal patterns transformation and Isolation Forest
2018	[101]	Traffic Flow Features as Metrics (TFFM)
2021	[102]	ML-based Anomaly Detection for resource-constrained IoT devices
2023	[103]	Statistical–Fog computing
2023	[104,105]	Deep Learning—Autoencoder and Neural Network
2023	[106]	Deep Learning—CNNs (Convolutional Neural Networks)
2023	[107]	Deep Learning—Unsupervised
2020	[108]	Empirical Data Analysis (EDA) and Gaussian kernel
2020	[109]	ML—semi-supervised
2021	[110]	Intrusion Detection System (IDS)
2023	[111]	Swarm Intelligence (SI)

**Table 8:** Signature-based detection approaches.

Year(s)	Paper(s)	Detection Technique Features
2017	[114]	Model-based testing and policy-based management
2020	[113]	Mirai traffic signatures
2022	[115]	Interpolation reasoning
2022	[116]	Firewall rules

**Table 9:** P2P-based detection approaches.

Year(s)	Paper(s)	Detection Technique Features
2019	[117]	Collect traffic metrics
2019	[118]	Mix with blockchain
2020	[119]	Multi-agent system
2023	[118]	Intelligent agent-based and ML

**Table 10:** Blockchain-based detection techniques.

Year(s)	Paper(s)	Detection Technique Features
2019	[120]	Collaborative Blockchain-Based Detection
2018	[121]	AutoBotCatcher: Blockchain-based P2P Botnet Detection
2019	[122]	Blockchain with SDN to prevent IOT botnets
2021	[123]	Blockchain with Proof-of-Contribution Mechanism
2022	[124]	Ethereum Blockchain Technology
2022	[125]	Blockchain-Enabled Secure Digital Twin Framework
2022	[126]	Blockchain-based Intrusion Detection System
2022	[127]	Blockchain with Machine Learning Intrusion Detection System

**Table 11:** ML-based detection techniques.

Year(s)	Paper(s)	Detection Technique Features
2021	[128,129,130]	Support Vector Machine (SVM)
2020–2021	[128,129,130]	Decision Tree
2020–2021	[128,129,130]	Random Forest
2022	[129,131]	K-Nearest Neighbor (KNN)
2022	[129,132]	Gradient Boosting (GB) (Decision Tree)
2021	[128]	Principal Component Analysis (PCA)

**Table 12:** DL-based detection techniques.

Year(s)	Paper(s)	Detection Technique Features
2019	[134]	Hybrid Learning
2020	[130,135]	Neural Network
2023	[133,136]	CNN
2023	[132]	Ensemble Learning
2023	[136]	LSTM

## Data Availability

No new data were created or analyzed in this study. Data sharing is not applicable to this article.
